# Emergent Spin Frustration in Neutral Mixed-Valence 2D Conjugated Polymers: A Potential Quantum Materials Platform

**DOI:** 10.1021/jacs.2c11185

**Published:** 2023-03-06

**Authors:** Isaac Alcón, Jordi Ribas-Ariño, Ibério de P.R. Moreira, Stefan T. Bromley

**Affiliations:** † Catalan Institute of Nanoscience and Nanotechnology (ICN2), CSIC and BIST, Campus UAB, Bellaterra, 08193 Barcelona, Spain; ‡ Departament de Ciència de Materials i Química Física & Institut de Química Teòrica i Computacional (IQTC), Universitat de Barcelona, c/ Martí i Franquès 1-11, 08028 Barcelona, Spain; § Institució Catalana de Recerca i Estudis Avançats (ICREA), Passeig Lluís Companys 23, 08010 Barcelona, Spain

## Abstract

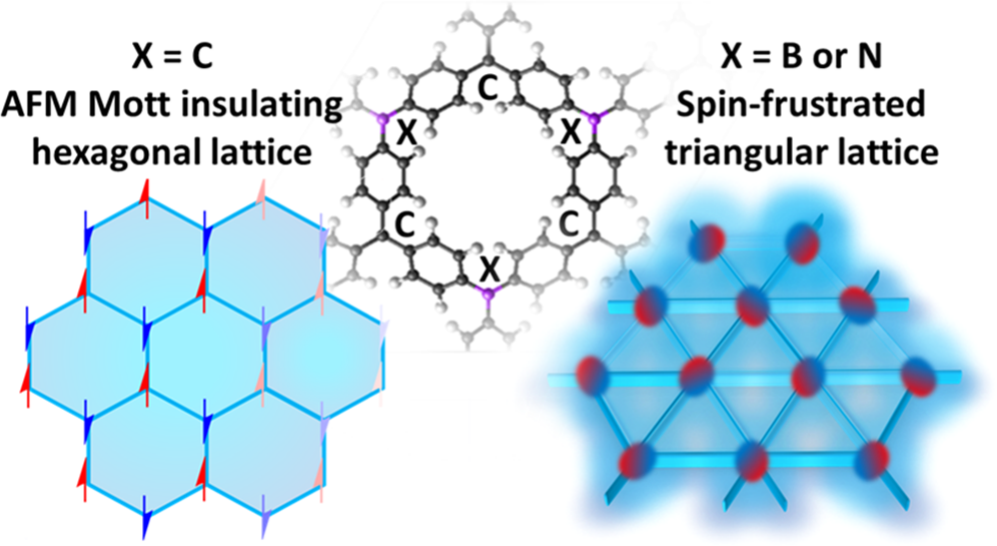

Two-dimensional conjugated polymers (2DCPs)—organic 2D materials composed of arrays of carbon sp^2^ centers connected by π-conjugated linkers—are attracting increasing attention due to their potential applications in device technologies. This interest stems from the ability of 2DCPs to host a range of correlated electronic and magnetic states (e.g., Mott insulators). Substitution of all carbon sp^2^ centers in 2DCPs by nitrogen or boron results in diamagnetic insulating states. Partial substitution of C sp^2^ centers by B or N atoms has not yet been considered for extended 2DCPs but has been extensively studied in the analogous neutral mixed-valence molecular systems. Here, we employ accurate first-principles calculations to predict the electronic and magnetic properties of a new class of hexagonally connected neutral mixed-valence 2DCPs in which every other C sp^2^ nodal center is substituted by either a N or B atom. We show that these neutral mixed-valence 2DCPs significantly energetically favor a state with emergent superexchange-mediated antiferromagnetic (AFM) interactions between C-based spin-^1^/_2_ centers on a triangular sublattice. These AFM interactions are surprisingly strong and comparable to those in the parent compounds of cuprate superconductors. The rigid and covalently linked symmetric triangular AFM lattice in these materials thus provides a highly promising and robust basis for 2D spin frustration. As such, extended mixed-valence 2DCPs are a highly attractive platform for the future bottom-up realization of a new class of all-organic quantum materials, which could host exotic correlated electronic states (e.g., unusual magnetic ordering, quantum spin liquids).

## Introduction

Two-dimensional conjugated polymers (2DCPs) are based on planar networks of trigonal sp^2^ nodes and are an emerging class of organic materials with a wide range of potential device applications.^1−8^ Graphene,^9^ a 2D hexagonal array of carbon sp^2^ nodes, is the most celebrated 2DCP largely due to its semimetallic band structure with Dirac-like linear band crossings at the Fermi level (*E*_F_), and concomitant high electron mobilities.^10^ However, as graphene does not display a band gap, it is not a suitable platform for switchable electronic devices. Theoretically proposed hexagonal 2DCPs based on modifying and extending the links between graphene’s sp^2^ nodes (e.g., 2D covalent organic radical frameworks (2D-CORFs), graphynes) were initially also predicted to be Dirac materials with corresponding graphenic electronic transport properties.^3,11,12^ Subsequently, however, a careful theoretical analysis of such 2DCPs showed that the semimetallic solution is metastable with respect to antiferromagnetic (AFM) multiradical or closed-shell quinoidal states.^13−17^ Synthesized 2DCPs have since been confirmed to exhibit AFM states.^18−20^ The associated finite band gaps of these states make 2DCPs potentially more attractive than graphene for electronic applications.

**Figure 1:**
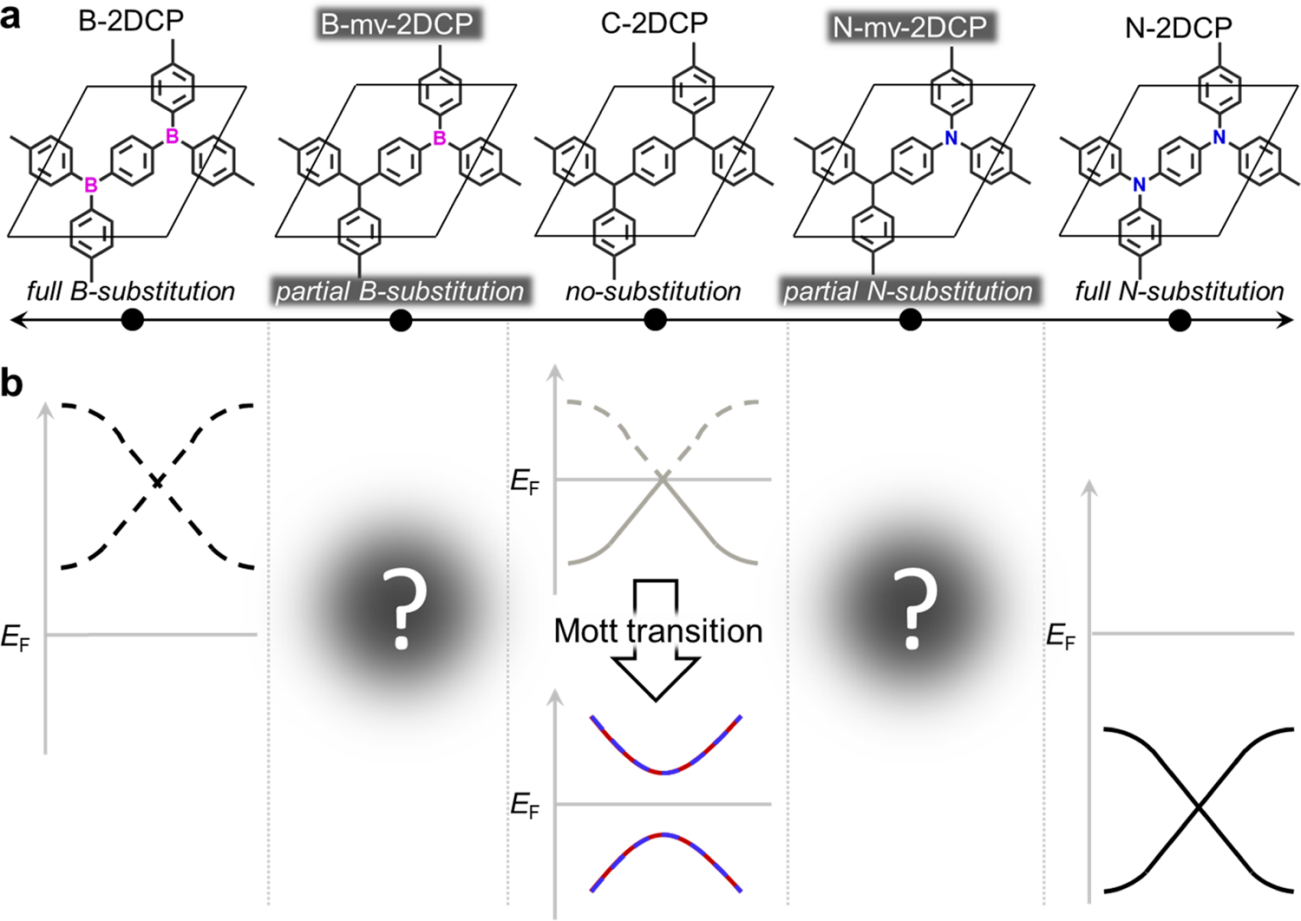
(a) Periodic structure of a series of chemically substituted 2DCPs. Note that hydrogen atoms are omitted in all structures for simplicity. The neutral mixed-valence 2DCPs (mv-2DCPs) considered in this work are highlighted. (b) Dependence of the electronic structure of 2DCPs on chemical substitution. Continuous and dashed lines represent occupied and conduction bands, respectively. EF denotes the Fermi level for conductors and an arbitrary energy level inside the gap for insulators. While both the nonsubstituted C-2DCPs and fully substituted situations (B- and N-2DCPs) have received much attention, the electronic structures of the partially substituted systems (i.e., B-mv-2DCP and N-mv-2DCP) are still unknown. See also Figure S1.

**Figure 2:**
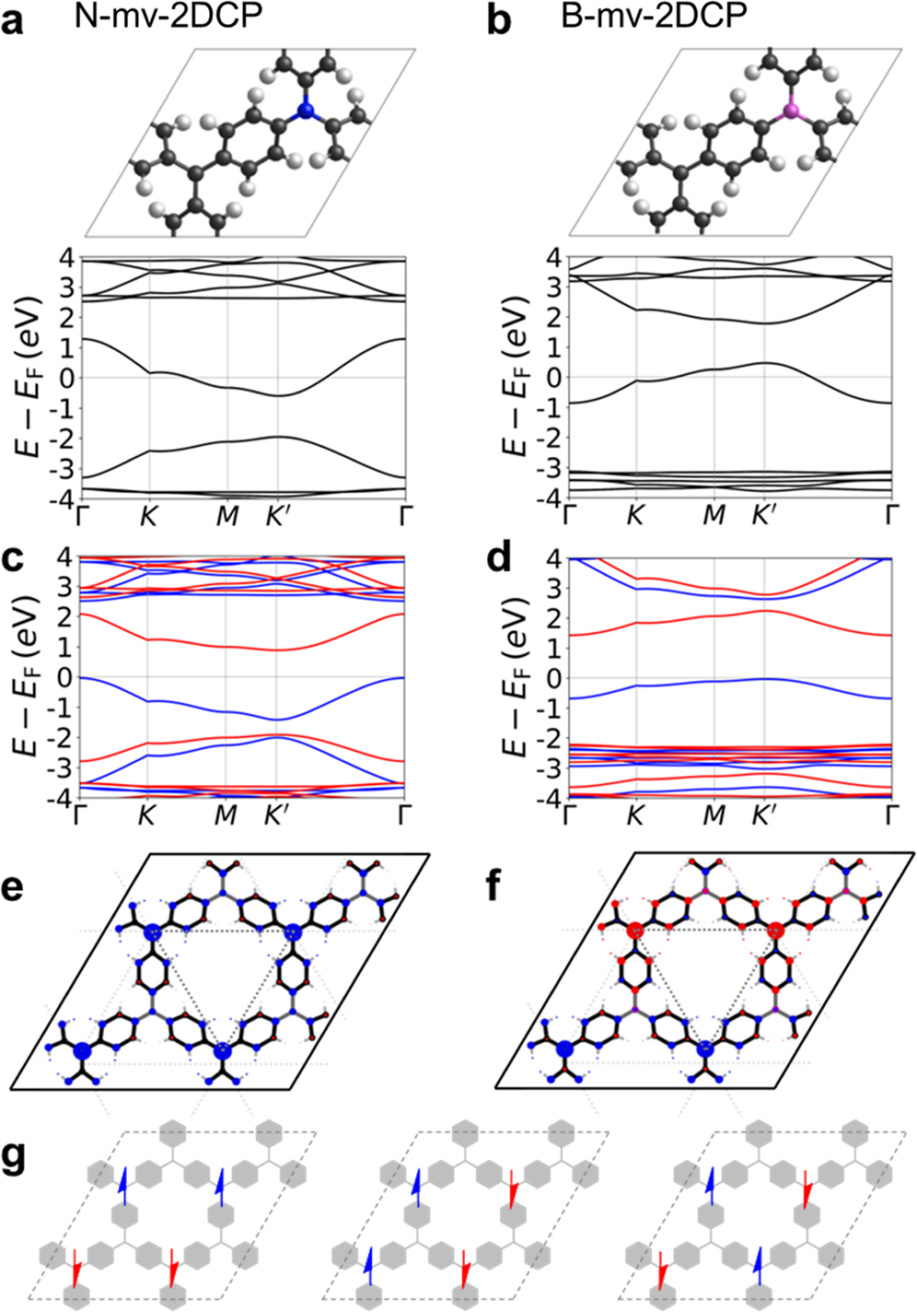
(a) Structure (top) and electronic band structure (bottom) for N-mv-2DCP and the same for (b) B-mv-2DCP, both from spin-restricted DFT calculations (i.e., diamagnetic solution). The respective unit cells contain one formula unit (fu). Atom color key: C—gray, H—white, B—mauve, N—blue. (c) Band structures for the ferromagnetic (FM) solution for the unit cell of N-mv-2DCP and for (d) B-mv-2DCP obtained from spin-unrestricted DFT calculations (spin-up: blue lines; spin-down: red lines). *E*_F_ has been set as the top of valence bands in (c,d). (e) Spin density map for the 2 × 2 supercell of N-mv-2DCP in the FM configuration and (f) an AFM configuration (spin-up: blue; spin-down: red). Note that the AFM alignment in (f) is not possible in the primitive cells shown in (a, b). Significant spin densities are only found on C sp^2^ nodes which form a highly symmetric triangular sublattice (dashed lines). (g) Representation of different near-degenerate AFM frustrated spin configurations in our mv-2DCPs.

The emergence of AFM states in hexagonal 2DCPs based on carbon sp^2^ centers (C-2DCPs) can be rationalized by considering these materials as lattices of singly occupied π-conjugated orbitals. Formally, the Hubbard effective Hamiltonian predicts such half-filled 2D systems to exhibit a Mott insulating AFM solution if the unpaired electrons on each node are sufficiently localized.^21^ Apart from very highly delocalized systems such as graphene, Mott-like AFM states are predicted to be typical for the more expanded, and thus more electronically localized, sp^2^ nodal networks of C-2DCPs.^12−16^ Chemical substitution of every carbon sp^2^ node in a C-2DCP with boron (B-2DCP) or nitrogen (N-2DCP) atoms removes or adds one electron per sp^2^ node, respectively. This either fills (N) or empties (B) the π-conjugated orbitals of the substituted 2DCPs networks such that the half-filling requirement for a Mott AFM insulating phase is not fulfilled. Consequently, as schematically illustrated in Figure 1, B-2DCPs and N-2DCPs are insulators that have been shown to recover the linear band crossings (i.e., Dirac cones) above and below *E*_F_, respectively.^22^ This behavior is in line with theoretical predictions^3^ and has also been experimentally demonstrated.^4^

Although there have been significant efforts to understand fully substituted B-, N-, and C-2DCPs, to the best of our knowledge, there are no reports on the electronic and magnetic properties of the corresponding neutral mixed-valence 2DCPs (mv-2DCPs). Such materials could be formed by periodically combining carbon nodes with either nitrogen or boron nodes, as depicted in Figure 1a. For C-2DCPs, the low-energy AFM and dimerized electronic solutions have strong analogies with known valence bond forms of molecular systems such as Thiele’s hydrocarbon,^23,24^ which may be thought of as a triarylmethyl^25^ (TAM) dimer. Similarly, insights into the likely electronic properties of mv-2DCPs systems can be gained by considering the analogous neutral mixed-valence molecular systems. For instance, linking a TAM and a triarylamine (TAA) via a π-conjugated linker results in neutral C–N mixed-valence compounds,^26−28^ which host a localized unpaired electron on the sp^2^ carbon center.^29^ These types of compounds have been shown to act as donor–acceptor systems that may be optically excited to undergo N-to-C charge transfer processes.^27^ Mixed-valence diradicals, triradicals, and even polymers made of alternating TAMs and TAAs displaying similar excitations have also been synthesized.^30,31^ Similarly, in diradicals or triradicals where the TAM centers are bridged by triarylborane (TAB) centers, optical excitation results in C-to-B charge transfer processes.^30^ All of these characteristics, which are not exhibited in the corresponding unmixed molecular systems (i.e., pure TAM, TAA, or TAB dimers), strongly suggest that extended sp^2^-based mv-2DCPs could exhibit new exotic electronic and magnetic phenomena.

Herein, we investigate the electronic properties that emerge when expanding the concept of neutral mixed-valence molecules to extended mv-2DCP materials. Specifically, we use first-principles density functional theory (DFT)-based calculations to study N-mv-2DCP and B-mv-2DCPs (see Figure 1a). Our study reveals that mv-2DCPs display well-localized spin centers on their carbon sp^2^ nodes, in full agreement with their molecular analogues.^29^ The magnetic interactions between spins on the radical carbon atoms are found to have an AFM character. However, the triangular symmetry of the carbon sublattice prevents extended 2D AFM ordering due to spin frustration. By modeling the analogous mv-diradical, mv-triradical, and mv-cyclo-triradical molecules, we find that such AFM interactions have a local character and have a strength that should ensure that the magnetic interactions are robust at room temperature. By comparing the mv-cyclo-triradical molecules with an isoelectronic electron-doped carbon-based hexamer, we find that spin frustration cannot be obtained in the parent C-2DCPs by, for instance, electrochemical reduction. This highlights the unique spin-electronic nature of our proposed mv-2DCPs. By *ab initio* molecular dynamics (AIMD) simulations, we also find that the spin-polarized nature of these materials is persistent to thermally induced structural fluctuations, contrary to C-2DCPs, where the spin-electronic structure is more sensitive to structural conformation.^15,16^ The magnetic interactions in our materials depends on the sterically determined twist angles of the linking aryl rings in these mv-2DCPs networks.^32^ These twist angles can, however, be modified by chemical functionalization and/or external strain/pressure^15,16,33,34^ to tune the AFM interaction strength.

The fact that we find robust AFM interactions between sites on an emergent triangular network in mv-2DCPs could be of particular interest due to their potential for hosting exotic quantum states due to the frustrated spin–spin interactions nature of such systems. The covalent skeleton of mv-2DCPs provides a rigid and highly symmetric 2D system in which structural distortions to remove spin frustration thus are strongly inhibited. The quantum states of materials in which localized *S* = ^1^/_2_ spins on a fixed lattice interact by isotropic spin exchange coupling are often analyzed in terms of the Heisenberg Hamiltonian. On the frustrated 2D triangular lattice, this model is predicted to have an ordered AFM-like insulating ground state.^35^ Generally, such frustrated magnetic systems can host a wide variety of unusual ordered phases with complex dynamics upon increases in temperature.^36^ Predictions of the pure Heisenberg model may also be affected by spin–orbit coupling, which can lead to spin canting competing with frustration via Dzyaloshinskii–Moriya interactions.^37^ The lack of significant spin–orbit coupling in organic sp^2^-based systems would tend to rule out noncollinear canted spin states in our mv-2DCPs. Specifically, we note that electron paramagnetic resonance measurements on the molecular analogues of both B-mv-2DCP and N-mv-2DCP can be well fitted using the Bleaney–Bowers equation, which is purely based on a two-body Heisenberg model (i.e., isotropic spin exchange coupling).^30^ Moreover, the *g*-factors in triphenylmethyl radicals have very similar values to that of the free electron, again confirming the almost total absence of any spin–orbit coupling effect.^38^

Formally, the Heisenberg model also corresponds to the Hubbard model in the limit of a large *U*/*t* ratio, where *U* is the onsite Coulomb repulsion term and *t* is the intersite hopping integral (i.e., the Mott insulating regime). The parent nonsubstituted C-2DCP material is a Mott insulator with AFM spin ordering on the hexagonal 2D lattice. However, it can be tuned toward a semimetal–insulator transition (i.e., lowering the *U*/*t* ratio) by chemical functionalization and out-of-plane compression.^15^ In the case of our proposed mv-2DCPs, as a Mott insulator they could likely host various spin-ordered phases on the frustrated 2D triangular lattice. Around the metal–insulator transition, quantum spin liquid (QSL)-based states have been formally predicted to emerge on 2D triangular lattices of *S* = ^1^/_2_ spins.^39,40^ Mechanically and/or chemically tuning the *U*/*t* ratio toward the (semi)metal–insulator transition could also open up the possibility of QSLs in our materials.

We note that ground-state predictions of model Hamiltonians are formally only valid at 0 K for a static unperturbed lattice and may not reflect the actual macroscopic observed state in materials at finite temperatures. The thermal stability of any spin-ordered or QSL-based state also depends on the strength of AFM coupling. In mv-2DCPs strong superexchange AFM interactions evolve between nearest-neighbor sp^2^ carbon centers forming a covalent triangular lattice of formally spin-^1^/_2_ centers. We find that the magnitude of the coupling strength (*J*) of the AFM interactions for our mv-2DCPs is comparable and even higher than those in well-known organometallic materials exhibiting a QSL character. Overall, our study provides a general design recipe to realize spin frustration in carbon-based 2D materials by embedding a triangular lattice of spin-^1^/_2_ centers in mv-2DCPs. We thus suggest that mv-2DCPs could provide a promising all-organic 2D quantum materials platform for exploring exotic magnetically ordered phases and even superconducting or QSL-based states.

## Results and Discussion

We evaluate the structure and properties of our mv-2DCPs with periodic DFT calculations using the hybrid PBE0 functional,^41^ which has been shown to properly capture the electronic structure of this type of 2D material^13^ and their molecular analogues.^15,16,42^ Closed-shell states were obtained via spin-restricted calculations, whereas the open-shell solutions were generated via spin-unrestricted electronic configurations, using spin-polarized initial guesses for each particular case (see the Methods Section). Here, we consider mv-2DCPs based on sp^2^ nodes, which are connected via single benzene rings in the hexagonal lattice making up their periodic structure (see Figure 1a). We note that macro cycles composed of six sp^2^ nodes with such type of ring-sharing connectivity have been synthesized based on C,^42^ N,^43^ and B–N^44,45^ and C–N^46^ combinations but not, as far as we are aware, on C–B combinations.

Figure 2a,b shows the optimized structures of our considered mv-2DCPs, namely, the N- and B-mv-2DCPs. We note that in each case, the *a* and *b* parameters are equal with an angle of almost exactly 60° indicating that the B and N substitutions negligibly affect the original hexagonal symmetry of the parent C-2DCP. In a spin-restricted configuration, partial filling (emptying) of the conduction (valence) bands occurs and N- and B-mv-2DCPs are predicted to be metallic and diamagnetic. However, allowing the system to break the spin degeneracy using a spin-unrestricted setup, one obtains a spin-polarized solution (i.e., paramagnetic) emerging from the C sp^2^ nodes for both mv-2DCPs as shown with the associated spin density maps for N- (Figure 2e) and B-mv-2DCP (Figure S2a in the Supporting Information, SI). In this situation, the previously partially filled bands split into a fully filled spin-up channel and an empty spin-down channel (see Figure 2c,d for N- and B-mv-2DCP). These spin-polarized gapped solutions are 167 meV and 287 meV per fu more stable than the spin-restricted diamagnetic metallic solutions for N- and B-mv-2DCP, respectively. This implies that the metallic solutions are energetically well above *k*_B_*T* at room temperature, and so almost inaccessible.

To assess the magnetic structure of our mv-2DCPs, we constructed a 2 × 2 in-plane supercell including four radical C sp^2^ centers for each case. Figure 2e shows a spin density map for N-mv-2DCP in the ferromagnetic (FM) configuration (see Figure S2 for B-mv-2DCP). Here, as the spin polarization emerges from the C sp^2^ centers, we obtain a regular triangular lattice of spin-^1^/_2_ centers, resulting from the N (B) chemical substitution in these networks. Figure 2f provides the analogous spin density map for an AFM configuration which, as shown in Figure S2, may also be obtained for B-mv-2DCP. Importantly, the AFM solution lies below the FM solution by 0.186 and 0.048 eV per fu for N- and B-mv-2DCP, respectively. This means that these mv-2DCPs significantly energetically favor emergent AFM solutions in which interactions between spin-^1^/_2_ centers create a regular triangular magnetic sublattice. This, in turn, inherently leads to magnetic frustration (i.e., where each C sp^2^ center cannot be antiferromagnetically coupled with all its nearest-neighbor C sp^2^ centers simultaneously). This spin frustration also gives rise to the possibility of many distinct but energetically degenerate AFM-aligned spin configurations (see examples in Figure 2g). The associated density of states for the AFM and FM solution for N-mv-2DCP are shown in Figure S3.

The corresponding nearest-neighbor magnetic coupling constants between C sp^2^ centers of a standard Heisenberg–Dirac–Van Vleck spin Hamiltonian (with −*J*_*ij*_Ŝ*_i_*·Ŝ_*j*_ two-body interactions and *J*_*ij*_ < 0 denoting AFM interactions) have been evaluated by means of a mapping between the spin eigenstates of this spin model and the relevant electronic solutions with different spin arrangements (see the SI for full details). The calculated magnetic coupling constants are: *J* = −46 meV for N-mv-2DCP and *J* = −12 meV for B-mv-2DCP (see SI for full details). We note that the AFM interaction strength in N-mv-2DCP is predicted to be significantly larger than that found for the possible QSL candidates EtMe_3_Sb[Pd(dmit)_2_]_2_ (*J* = [−250,–220] K = [−22,–19] meV) depending on the susceptibility fitting^47,48^ and κ-(ET)_2_Ag_2_(CN)_3_ (with *J* = −220, −270, and −310 K, for *P* = 0.5, 0.75, and 0.95 GPa).^49^

**Figure 3:**
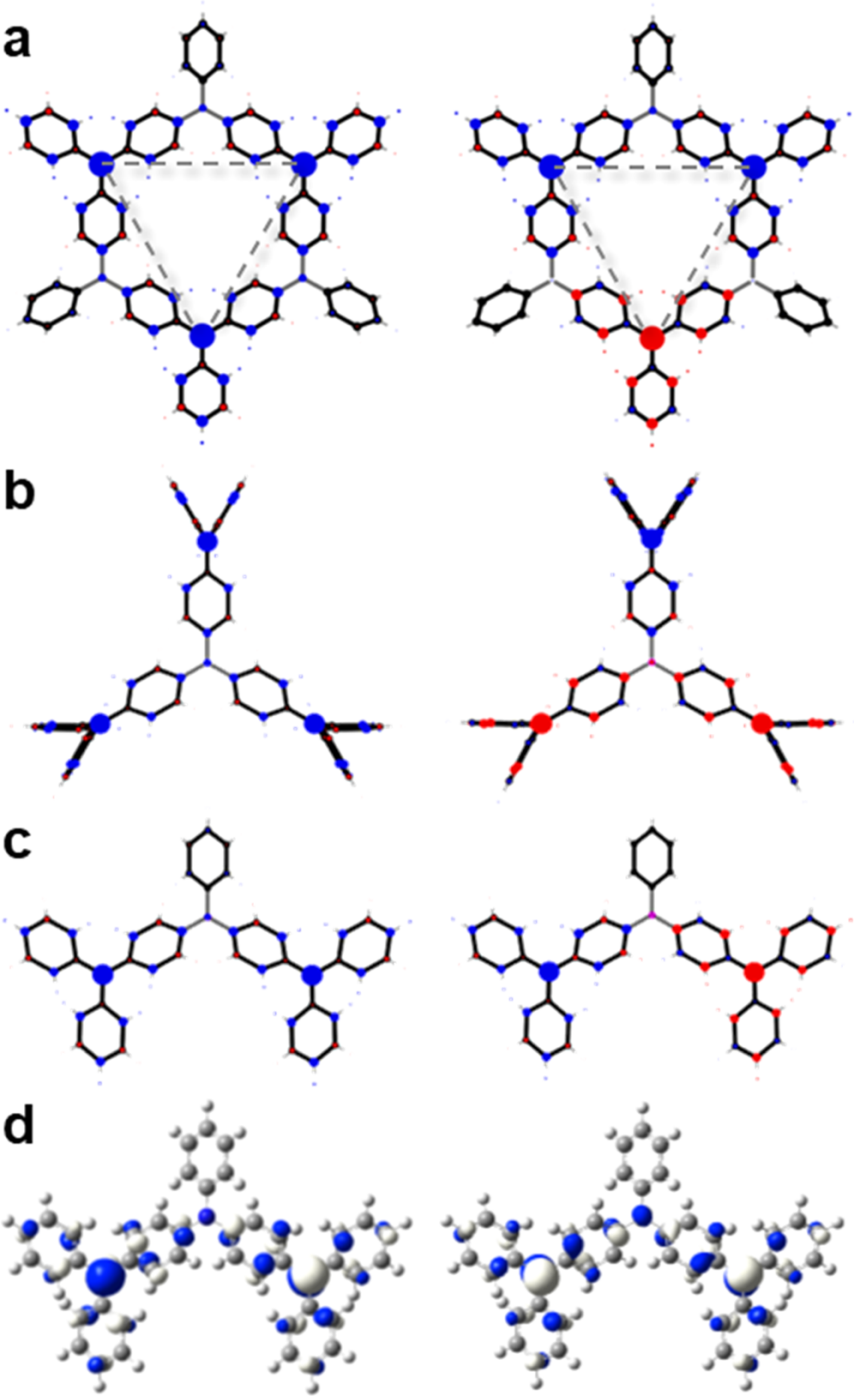
Spin density maps for (a) the CN-nanoring, (b) the CN model with three magnetic centers and (c) the CN model with two magnetic centers. FM spin configurations are shown on the left and AFM spin configurations are shown on the right. In (a–c), blue corresponds to spin-up and red corresponds to spin-down. (d) Singly occupied natural orbitals (SONO) for the singlet ground state of the CN model with two magnetic centers. These orbitals were obtained from unrestricted DFT calculations carried out with the Gaussian 09 program^50^ using the PBE0 functional and an Ahlrichs TZVP basis set.^51^ The electron occupation is 1.181 and 0.819 for the antisymmetric (left) and symmetric (right) SONOs, respectively.

To better understand the type of magnetic interactions in these mv-2DCPs, we now consider their molecular analogues. We note that the synthesis of molecular neutral mixed-valence systems of the type we consider below has been shown to be feasible and to provide a good platform for studying magnetic interactions.^30^ In Figure 3a, we consider CN-based nanoring molecular analogue of our extended N-mv-2DCP where we can obtain both the FM and AFM solutions. This is also the case for the CB-based nanoring molecular analogue of the B-mv-2DCP (see Figure S4). As in the corresponding mv-2DCPs, the spin-frustrated AFM configuration is the ground state for these molecular systems, being 53 meV (16 meV) per fu below the FM solution for the CN-based (CB-based) nanorings. This strongly suggests that the magnetic interactions in both molecular and extended systems arise at the local scale. This is further corroborated by considering even smaller molecular analogues, including two or three magnetic centers, also displaying an AFM ground state (see Figures 3b,c and S4 for the corresponding Δ*E*_AFM-FM_ values). These results are also consistent with those reported elsewhere.^30^ We note that the magnitude of the *J* coupling constants of our considered molecular systems is larger than those in ref (30) due to (i) the more planar conformation of aryl rings in our systems compared to those based on perchlorotriarylmethyls, and (ii) the fact that our sp^2^ nodes are separated only by a single aryl ring, as opposed to two aryl rings. We also note that a triangular arrangement of AFM coupled spin-^1^/_2_ centers is not obtained when the analogous full-carbon nanoring (as recently synthesized^42^) is triply reduced, as shown in Figure S5, despite the fact it is isoelectronic with the CN-based nanoring. This highlights the need for chemical substitution, as herein proposed, to induce spin frustration either in the molecular systems or in the corresponding 2D materials.

The nature of the magnetic interactions in mv-2DCPs can be further understood by close inspection of the frontier orbitals of the corresponding molecular models including two magnetic centers. The singly occupied natural orbitals (SONOs) of the CN and CB models with two magnetic centers show that the unpaired electrons are mainly localized on the TAM moieties (i.e., the carbon sp^2^ nodes). Specifically, the SONOs result from the symmetric and antisymmetric combinations of the singly occupied molecular orbitals (SOMOs) of the TAMs and a small contribution of the p orbitals of the bridging B/N atom (see Figures 3d and S6). Both the CN (Figures 3d) and CB models (Figure S6) with two magnetic centers have a SONO with an occupation significantly larger than 1.0 and a SONO with an occupation significantly smaller than 1.0. In the case of the CN model with two magnetic centers, the SONO with a larger occupation Figure 3d, left) does not involve any contribution of the p orbital of the N atom. However, for the SONO with a smaller occupation (Figure 3d, right) , the p orbital of N combines in an antibonding fashion with the p orbitals of its neighboring atoms (see Figure 3d). In the case of the CB models with two magnetic centers, the SONO with a larger occupation features a bonding combination of the p orbital of B with the p orbitals of its neighboring atoms, whereas the SONO with a smaller occupation does not involve any contribution of the p orbital of the B atom (see Figure S6). The differences in the atomic contributions of the SONOs of the CN and CB models can be rationalized by means of a simple molecular orbital diagram, as shown in the SI (Figure S12). The shape of the SONOs suggests that complex electronic interactions between the localized unpaired electrons through the bridging π-system in N- and B-mv-2DCP is similar to a superexchange mechanism where the π-orbitals (and electrons) of the central N or B atoms play the role of the closed-shell ligand in Anderson model.^52,53^ This helps to rationalize the large AFM interactions found in these systems and its dependence on structural deformations (see below).

**Figure 4:**
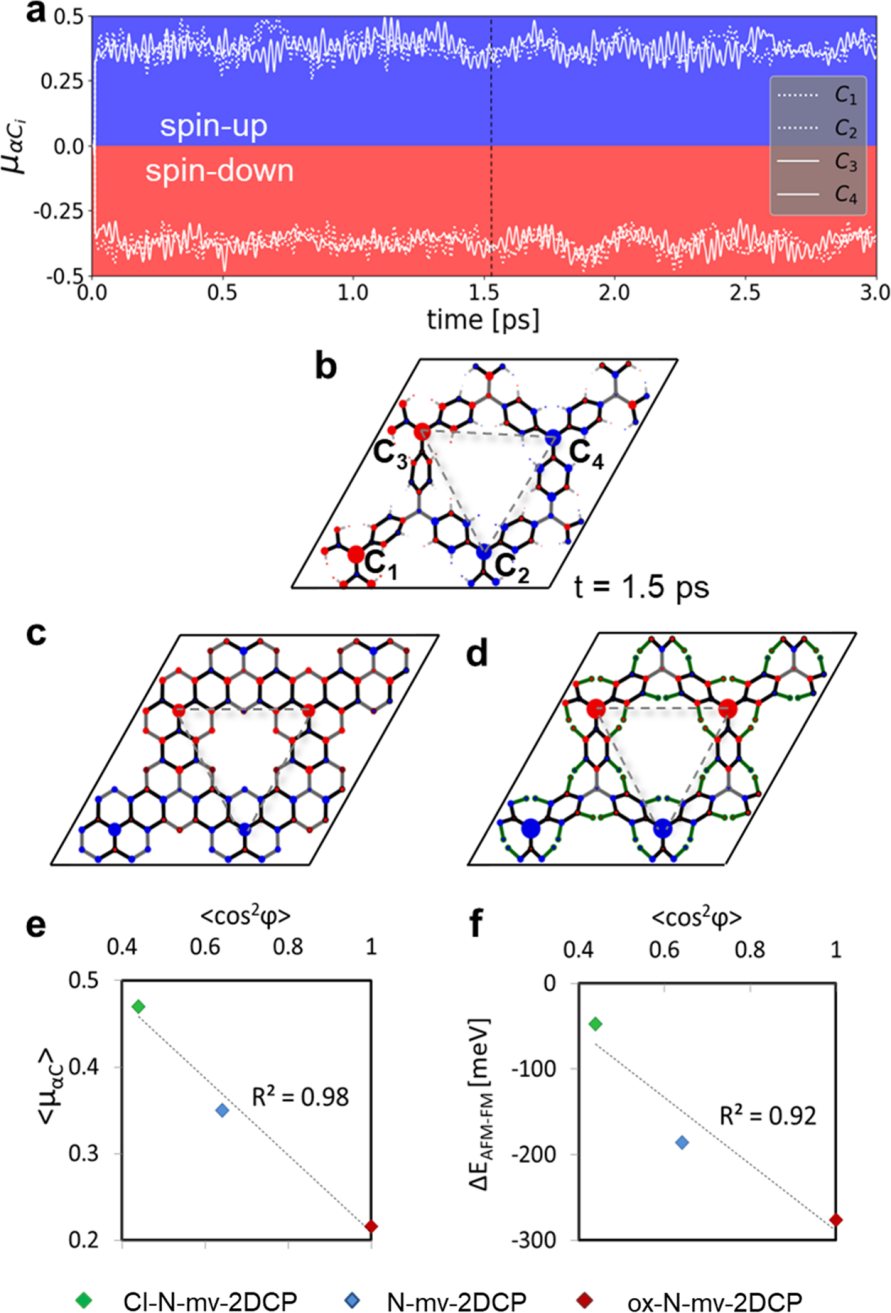
(a) Spin populations on each α*C* center (see (b)) in N-mv-2DCP (μ_*αC*_*i*__) during a 3 ps AIMDS at 300 K. (b) Spin density maps for an N-mv-2DCP taken from a snapshot during the AIMD calculations at the time indicated with vertical dashed line in (a). (c) Spin density maps for the fully optimized ox-N-mv-2DCP and for (d) Cl-N-mv-2DCP structures in an AFM spin configuration (see the Methods Section for details). (e) Average of the absolute value of spin population on the αCs (⟨|μ_α*C*_|⟩) against ⟨cos φ_*i*_^2^⟩ for the three different considered N-mv-2DCPs in (b–d). ⟨cos φ_*i*_^2^⟩ = ∑ cos φ_*i*_^2^ / *n* and ϕ*_i_* are the twist angle of each aryl ring *i* with respect to the 2D material plane. (f) Energy difference between the AFM and FM configurations against ⟨cos φ_*i*_^2^⟩ for the same three 2DCPs.

As previously shown for the parent C-2DCPs, the conformation of the 2D framework (e.g., dihedral angles of aryl rings) can determine its electronic configuration.^15,16^ For instance, the effect of thermal fluctuations may lead to significant variations in the total absolute spin population of the networks, due to electron pairing.^16^ On the other hand, the chemical functionalization of aryl rings also affects the multiradical character of C-2DCPs, as it affects the π-conjugation via the dihedral twist angles of the aryl rings.^33,34^ To evaluate the impact of these different parameters, we first used ab initio molecular dynamics (AIMD) at 300 K for N-mv-2DCP (see the Methodology Section for details). Here, we set the initial spin polarization to zero to avoid any bias towards an AFM solution, thus allowing the system to spontaneously evolve with temperature. As shown in Figure 4a, a rapid spontaneous spin population appears for each of the four αCs within the unit cell which couple via local AFM interactions (i.e., two μ_α*C*_*i*__ > 0 and two μ_α*C*_*i*__ < 0). We can see the resulting AFM configuration more clearly by examining the spin density map shown in Figure 4b for a random snapshot during the dynamics. We can also see that this AFM configuration remains stable for the full 3 ps of our AIMD run, with no significant drops in the average of the absolute value of the α*C* spin populations (⟨|μ_α*C*_|⟩; see Figure S7). This result also highlights the different nature of N-mv-2DCP (and B-mv-2DCP) compared to the analogous C-2DCP, where ⟨|μ_α*C*_|⟩ is significantly lower in magnitude and varies strongly during an AIMD run. In fact, in C-2DCP, ⟨|μ_α*C*_|⟩ can sometimes completely vanish due to the possibility of electron pairing of radical centers forming a quinoidal configuration.^16^ Such a pairing mechanism is not observed for N-mv-2DCP and B-mv-2DCP, as the N and B substitutions inhibit direct interactions between C centers. Consequently, all αC centers exhibit a robust associated spin-^1^/_2_, thus equipping the resulting 2D frameworks with a persistent multiradical character which is undisturbed by thermally induced structural fluctuations.

Finally, we considered the effect of chemical functionalization of the aryl rings in our mv-2DCPs. The localization of spin density depends on the dihedral angle of the aryl rings^34^ which, in turn, affects the magnetic interactions between spin-^1^/_2_ radical centers in the 2D frameworks.^32^ Here, we consider two additional N-mv-2DCPs built from the molecular oxygen-functionalized triarylmethyl (oxTAM)^54^ and perchlorotriarylmethyl (PTM).^55^ These molecular functionalizations lead, respectively, to fully planarized aryl rings within the ox-N-mv-2DCP, ⟨φ⟩ = 0.02°, and to highly out-of-plane twisted aryl rings for the Cl-N-mv-2DCP, ⟨φ⟩ = 48.5° deg., compared with the hydrogenated N-mv-2DCP, ⟨φ⟩ = 36.8° (see Figure S8). As shown in Figure 4, both the ox-N-mv-2DCP (4c) and Cl-N-mv-2DCP (4d) also display an AFM ground state, being −276 and −47 meV per fu below the respective FM solution. The corresponding magnetic coupling constants between nearest-neighbor sp^2^ centers are calculated to be: *J* = −68 meV (ox-N-mv-2DCP) and *J* = −13 meV (Cl-N-mv-2DCP).

These results demonstrate that the AFM interactions are inherently correlated with the alternating distribution of C and N (B) sp^2^ nodes within N-mv-2DCPs (B-mv-2DCPs). The chemical functionalization of aryl rings, though, has a significant effect on the strength of such magnetic interactions. In Figure 4e, we plot ⟨|μ_α*C*_|⟩, which gives a measure of spin localization, against the average of the cosine squared of all aryl rings twist angles (⟨cos φ_*i*_^2^⟩). As we may see, there is a linear correlation between the two quantities, which is in line with previous studies,^32,34^ and highlights the principal role of aryl ring twist angles to determine spin localization in this type of 2DCPs. Since spin localization has a direct impact on magnetic interactions, we also obtain a correlation between Δ*E*_AFM-FM_ and ⟨cos φ_*i*_^2^⟩ (see Figure 4f). These results highlight the role of the chemical functionalization of aryl rings to tune the strength of the spin-frustrated magnetic phase in mv-2DCPs. The highly twisted aryl rings in Cl-N-mv-2DCP (see Figure S8) leads to strong spin localization (Figure 4d), which entails a smaller AFM coupling (see above). However, the fully planarized ox-N-mv-2DCP has significant spin delocalization and we obtain a larger AFM coupling (see above), which should be robust even at room temperature (Figure 4c). These trends can be understood in terms of the Anderson model since a larger planarity increases orbital delocalization through the bridging aromatic rings which, in turn, increases the superexchange contribution to the magnetic coupling to further stabilize the low spin states with respect to the high spin state.

As mentioned above, a 2D triangular lattice of strongly AFM interacting spin-^1^/_2_ centers can be described by the Heisenberg model for highly localized spins (i.e., for a Mott insulator with high onsite Coulomb repulsion, *U*). With increasing electron delocalization the hopping integral between sites (*t*) becomes more significant and tends to disrupt magnetically ordered states. The situation arising from *U*/*t* values corresponding to the metal–insulator transition is thought to be ideal for creating a QSL ground state where the system fluctuates between degenerate frustrated AFM spin configurations, even without thermal energy.^56−58^ For the Hubbard model on the 2D triangular lattice, the QSL regime has been predicted to occur for approximately 8 ≤ *U*/*t* ≤ 11.^39,40^ Following the approach in ref (59), we use the FM band structures of the three materials in Figure 4 to estimate their respective *U* and *t*.^59^ In line with the increasing delocalization with increasing planarity of the linkers going through the series: Cl-N-mv-2DCP, N-mv-2DCP, ox-N-mv-2DCP, we also find a concomitant increase in *t* and decrease in *U*. For Cl-N-mv-2DCP, the *U*/*t* value is estimated to be 35.5 which is deep inside the Mott insulating regime. However, for N-mv-2DCP and ox-N-mv-2DCP, the corresponding *U*/*t* values significantly decrease to 19.2 and 16.1 respectively (see Figure S10 in the SI). These extracted estimates of *U* and *t* are from all-electron calculations of realistic chemical structures and cannot thus be directly compared with the parameters used to define the approximate single-band Hubbard model. Nevertheless, they indicate that N-mv-2DCP and ox-N-mv-2DCP may be weak Mott insulators, i.e., close to the metal–insulator transition. Weak Mott insulators are thought to be particularly good candidates for hosting a QSL state.^56^ The chemically induced variability of *U*/*t* in our mv-2DCPs clearly demonstrates that modification of the spin-bearing units can be used to tune their electronic properties toward the QSL regime. The application of moderate out-of-plane pressure could also be used to tune the *U*/*t* ratio of these materials towards the metal–insulator transition.^15^ This is also in line with recent experiments carried out on a hybrid organic–inorganic molecule-based material showing that pressure can induce the emergence of a QSL state.^60^

Taken together, our results show that partially B- or N-substituted mv-2DCPs can host symmetric triangular lattices of AFM interacting spins for which the magnetic interaction strength can be tuned by chemical functionalization. If suitably tuned materials of this type were synthesized, the resulting frustration between different AFM configurations in mv-2DCPs could lead to the emergence of unusual spin-ordered states and potentially to elusive QSL-based states. The current most widely accepted candidate QSL-host materials are 2D organic salts such as κ-(ET)_2_Cu_2_(CN)_3_^49^ and EtMe_3_Sb[Pd(dmit)_2_]_2_,^47,48^ which form arrays of discrete molecules in a triangular lattice. These materials tend to have fairly low strength AFM interactions (*J* ∼−250 K = −21.5 meV) and their nonbonded arrays of molecular spin-carrying nodes are susceptible to thermal disruption due to the weak cohesive intermolecular forces taking place. In contrast, mv-2DCPs are based on robust and highly symmetric covalently linked sp^2^-based 2D lattice which can host significantly stronger AFM interactions. The low spin–orbit coupling in our sp^2^-based magnetic states also inhibits spin canting, which can lead to competing nonfrustrated spin orderings.^37^

Several experimental realizations of 2D spin lattice systems (e.g., square lattice in cuprates, the honeycomb lattice, or the frustrated triangular or kagomé lattices) have shown interesting quantum phases at low temperatures, such as superconductivity, QSL-based phases, or topological quantum states.^56,61−65^ Often the focus in these studies is on materials with lattices of antiferromagnetically interacting spins in which states exhibiting the conventional Néel order are thought to compete with resonating valence bond (RVB)-like states. It has been suggested that doped Mott insulators that are close to the (semi)metal–AFM–insulator transition could be good candidates to host such novel quantum phases. In relation to this, we note that our proposed B/N-substituted C-2DCPs could also be electrostatically doped by supporting them on an Au-based electrode. Such a setup has been shown to promote oxidation of N and C sites in 1D sp^2^-based systems.^66,67^ Perhaps the most spectacular example of electrostatic doping of a 2D material based on sp^2^ centers is realized in magic angle bilayer graphene, in which a transition between a Mott insulator and a superconducting phase was achieved.^68^ We further note that the redox properties (i.e., ease of doping) of our materials would also be tunable by chemical design.^30,69^ We thus envisage that electrostatic doping would be a feasible way to introduce additional spins in the triangular “2D Heisenberg sea” of mv-2DCPs in which the strong AFM interactions of mv-2DCPs would tend to induce large spin fluctuations. This scenario is much like that observed in the cuprates, for which the RVB theory of high-Tc superconductivity provides the most complete description of the pairing mechanism.^70^ We note that the magnitude of the magnetic coupling constants in our mv-2DCPs is comparable to those found in the parent compounds of superconducting cuprates (*J* ∼−125 meV = −1450 K) for La_2_CuO_4_,^71^ Nd_2_CuO_4_,^72,73^ and YBa_2_Cu_3_O_6_.^74^ In this sense, our proposed family of mv-2DCPs may provide the essential structural and electronic features necessary to develop a plausible chemical basis for a superconducting phase upon doping. In summary, we suggest that the properties of mv-2DCPs could have great potential for providing a well-defined, robust, and tunable general platform for developing a new class of all-organic sp^2^-based 2D quantum materials.

## Conclusions

We have studied the electronic and magnetic properties of a new class of 2DCPs by means of first-principles hybrid DFT calculations. Our proposed materials can be envisaged as chemically substituted versions of previously well-known hexagonal networks of sp^2^ carbon centers, here referred to as C-2DCPs (also known as 2D-CORFs). From another perspective, our materials can be conceptually viewed as proposed 2D extended versions of neutral mixed-valence molecules. The resulting N- and B-mv-2DCPs display a semiconducting multiradical ground state, which, contrary to the parent material (C-2DCP), is not in competition with closed-shell quinoidal configurations. This makes the spin-polarized multiradical state very robust against thermal fluctuations. Consequently, N- and B-mv-2DCPs may be regarded as 2D arrays of persistent spin-^1^/_2_ centers. We further find that the radical carbon nodes interact through significant AFM coupling in both N- and B-mv-2DCPs. These AFM interactions appear to be of a local character, as they also emerge for the corresponding molecular systems. Because of the extended triangular arrangement of spin-^1^/_2_ centers in these 2DCPs, such AFM coupling would lead to magnetic frustration, due to the impossibility of having all spin centers antiferromagnetically coupled with their nearest neighbors. Such a frustrated situation makes our proposed 2DCPs purely organic candidates to host unusual magnetically ordered states^36^ and, potentially, QSL-based states.^56−58,75^ We note that our strategy could also be applicable to other 2DCPs based on open-shell organic molecular building blocks e.g., triangulenes.^82^ Considering different chemical functionalizations of the aryl rings for N-mv-2DCP, we find that the strength of such (frustrated) AFM interactions may be tuned from ca. −50 to −300 meV, due to the effect of flattening aryl ring twist angles on spin localization. At the same time, the increase in delocalization leads to the estimated *U*/*t* ratio becoming progressively closer to the metal–Mott insulator transition, where QSL states are more likely to emerge. Due to the strong AFM coupling in some of our proposed materials (well above *k*_B_*T* at room temperature), it is likely that the magnetic frustration would be persistent at temperatures well above nitrogen liquid. Generally, our proposed mv-2DCPs are attractive synthetic targets for realizing a robust tunable all-organic quantum materials platform for bottom-up design and generation of exotic phases (e.g., QSL states, spin crystals, RVB states, superconductivity).

## Methodology

The atomic and electronic structure of our 2DCPs was modeled by first-principles density functional theory (DFT)-based calculations using periodic boundary conditions and the hybrid PBE0 functional.^41^ The PBE0 functional was found in prior studies to reproduce experimentally measured magnetic coupling coefficients of this type of systems.^16,42^ The closed-shell metallic solutions for N-mv-2DCP and B-mv-2DCP were obtained from spin-restricted DFT calculations. The two magnetic configurations (FM and AFM) were separately obtained by appropriately initializing the spin moment on αC radical centers in spin-unrestricted DFT calculations. Atomic coordinates and in-plane unit cell parameters were preoptimized using the PBE^76^ functional and a Tier-1 light numerical atom-centered orbital (NAO) basis set,^77^ as implemented in the Fritz Haber Institute *ab initio* molecular simulations package (FHI-AIMS).^78,79^ Subsequent full optimizations were performed with the PBE0 hybrid functional^41^ and the same NAO basis set. Optimizations of the unit cells (supercells) were performed using 18 × 18 × 1 (6 × 6 × 1) Γ-centered Monkhorst–Pack (MP) sampled **k**-grids. Convergence criteria of 1 × 10^–5^ eV and 1 × 10^–2^ eV/Å were used for the total energy and for the maximum atomic force component, respectively. The same functional, basis set, and convergence criteria were used for the studied molecular systems. Band structures, total energies, and atomically partitioned spin populations and spin densities were then extracted from single-point calculations using the fully optimized structures. These final SCF runs were performed using the PBE0 functional and the same MP **k**-grid and NAO basis set previously indicated for each periodic cell type. Atomically partitioned spin populations (μ*_i_*) were calculated using the Hirshfeld scheme.^80^ Finally, the effect of thermal fluctuations was modeled via ab initio molecular dynamics (AIMD) calculations at 300 K for 3 ps, using the Bussi–Donadio–Parrinello thermostat,^81^ the PBE0 functional, a 2 × 2 × 1 MP **k**-grid, and a Tier-1 light NAO basis set.

## Supporting Information Available

Electronic band structures for the B-, C- and N-2DCPs; atom-resolved spin population maps for FM and AFM configurations in the B-mv-2DCP; spin-resolved density of states for 2 × 2 supercells in N-mv-2DCP in FM and AFM configurations; atom-resolved spin population maps for FM and AFM configurations in all N- and B-based molecular systems; atom-resolved spin population maps for cyclo-para-phenylmethine; singly occupied natural orbitals for the B-based diradical molecule; time-resolved average of the αC spin population for N-mv-2DCP at 300 K; periodic structures of ox-N-mv-2DCP, N-mv-2DCP, and Cl-N-mv-2DCP; species-projected density of states for ox-N-mv-2DCP, N-mv-2DCP, and Cl-N-mv-2DCP; U/t estimation for ox-N-mv-2DCP, N-mv-2DCP, and Cl-N-mv-2DCP; magnetic coupling constant calculations; and molecular orbital diagrams for N- and B-based systems (PDF)
