# Supplementary material for: Emergent Spin Frustration in Neutral Mixed-Valence 2D Conjugated Polymers: A Potential Quantum Materials Platform
Source: J Am Chem Soc. 2023 Mar 6;145(10):5674–83. doi: 10.1021/jacs.2c11185 (PMC10021012; doi:10.1021/jacs.2c11185)
Supplement: Supplementary file 1 — ja2c11185_si_001.pdf [file ja2c11185_si_001.pdf]

Supporting Information for:

Emergent Spin Frustration in Neutral Mixed-Valence 2D Conjugated Polymers: A Potential Quantum Materials Platform

Isaac Alcón[1,*], Jordi Ribas-Arino[2], Ibério de P.R. Moreira[2] and Stefan T. Bromley[2,3,*]

[1]*Catalan Institute of Nanoscience and Nanotechnology (ICN2), Av. de Serragalliners, s/n, 08193 Bellaterra (Barcelona), Spain*

[2]*Departament de Ciència de Materials i Química Física & Institut de Química Teòrica i Computacional (IQTCUB), Universitat de Barcelona, c/ Martí i Franquès 1-11, 08028 Barcelona, Spain*

[3]*Institució Catalana de Recerca i Estudis Avançats (ICREA), Passeig Lluís Companys 23, 08010 Barcelona, Spain*

* Corresponding authors: isaac.alcon@icn2.cat, s.bromley@ub.edu

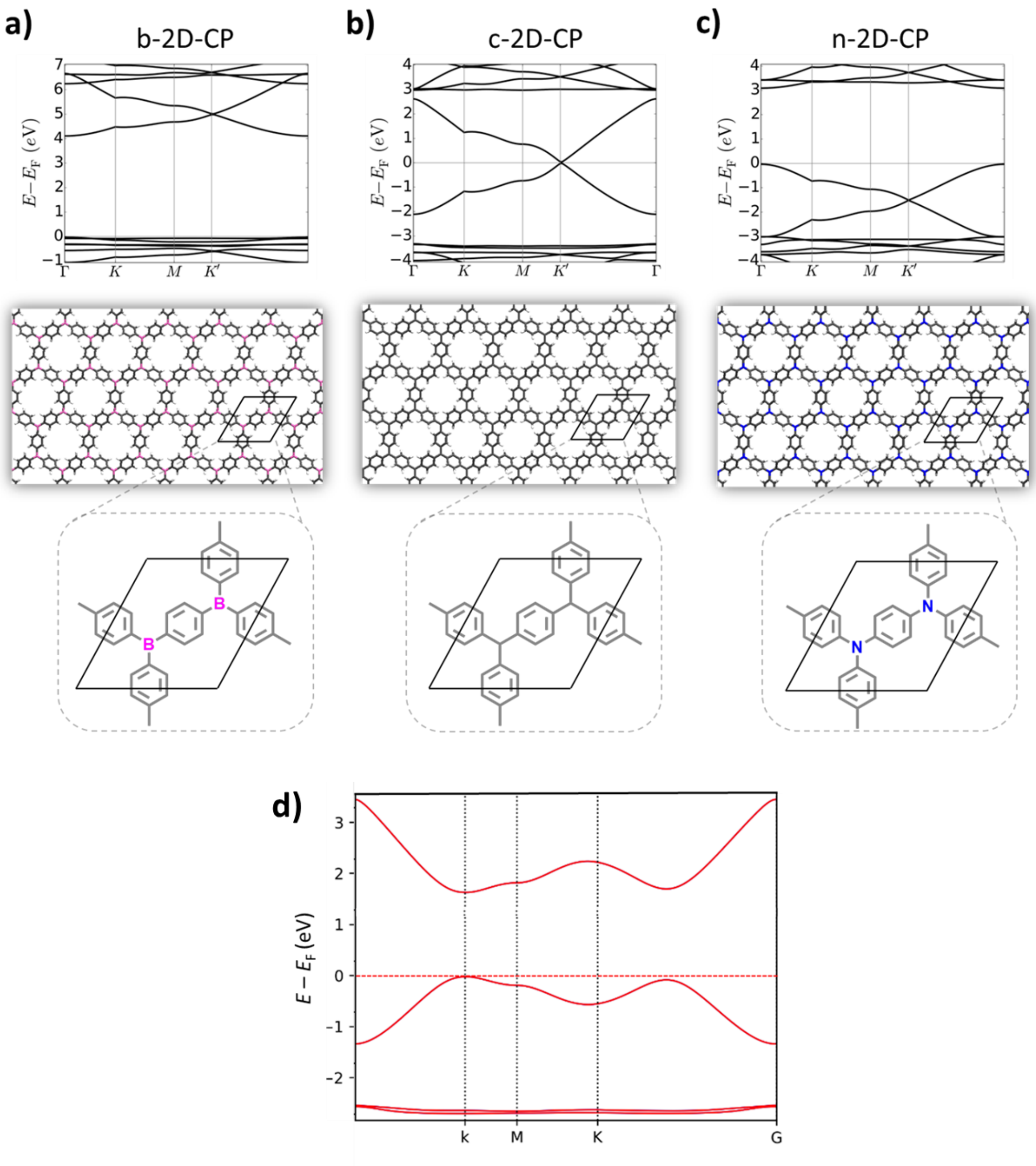

Fig. S1. Electronic band structures (top) and periodic atomic structures (bottom) for a) B-2D-CP, b) C-2D-CP, c) N-2D-CP (colouring: C – grey, H – white, B – mauve, N – blue), obtained from spin-restricted DFT calculations using the hybrid PBE0 functional. The chemical representation of the atomic periodic structure of each material omits hydrogen atoms. d) band structure of the AFM spin polarised solution of the C-2D-CP system which is lower in energy than the closed shell solution and shows a gap opening due to a Mott (semi)metal-to-insulator transition as discussed in the text (note that gapped AFM-like solutions are lower in energy than the FM solution – see discussion in the main text). E_F represents the Fermi level for conducting systems and an arbitrary energy in the insulating gap for insulators. It is set to the top of valence bands in the case of insulators.

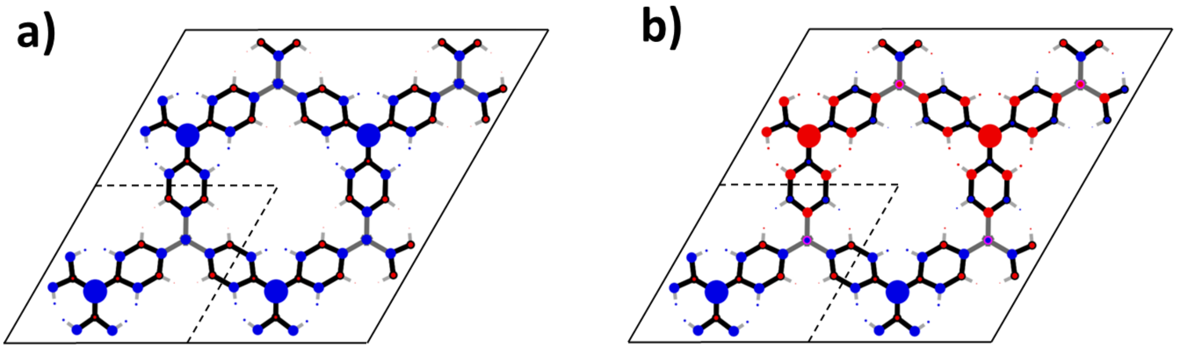

Fig. S2. Atomically-resolved spin population maps (spin-up: blue; spin-down: red) for B-mv-2DCP in a) the ferromagnetic (FM) and b) the antiferromagnetic (AFM) spin-polarized solutions.

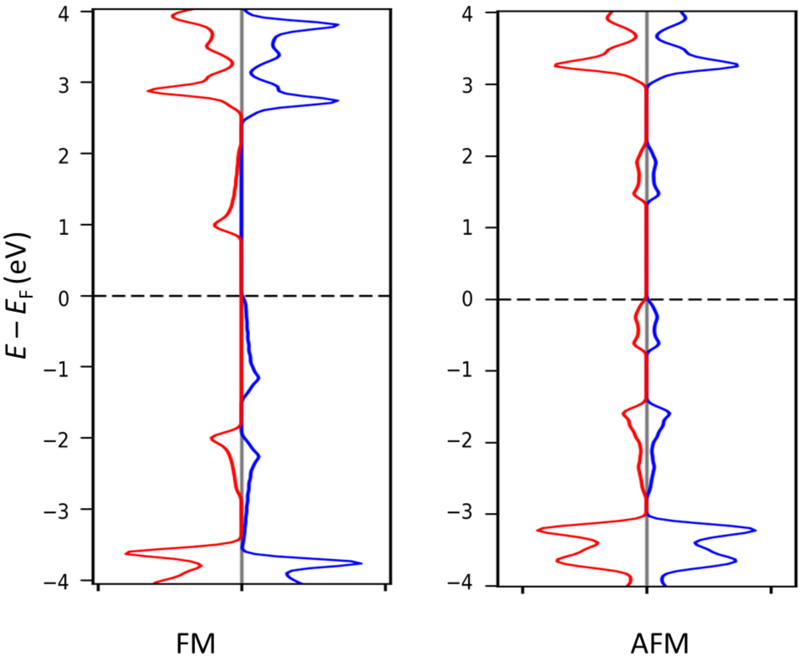

Fig. S3. Electronic density of states (DOS) plots for in the FM (left) and AFM (right) configurations for N-mv-2DCP. Spin-up: blue; spin-down: red. Energies are given with respect to E_F which is placed at the top of the valence band.

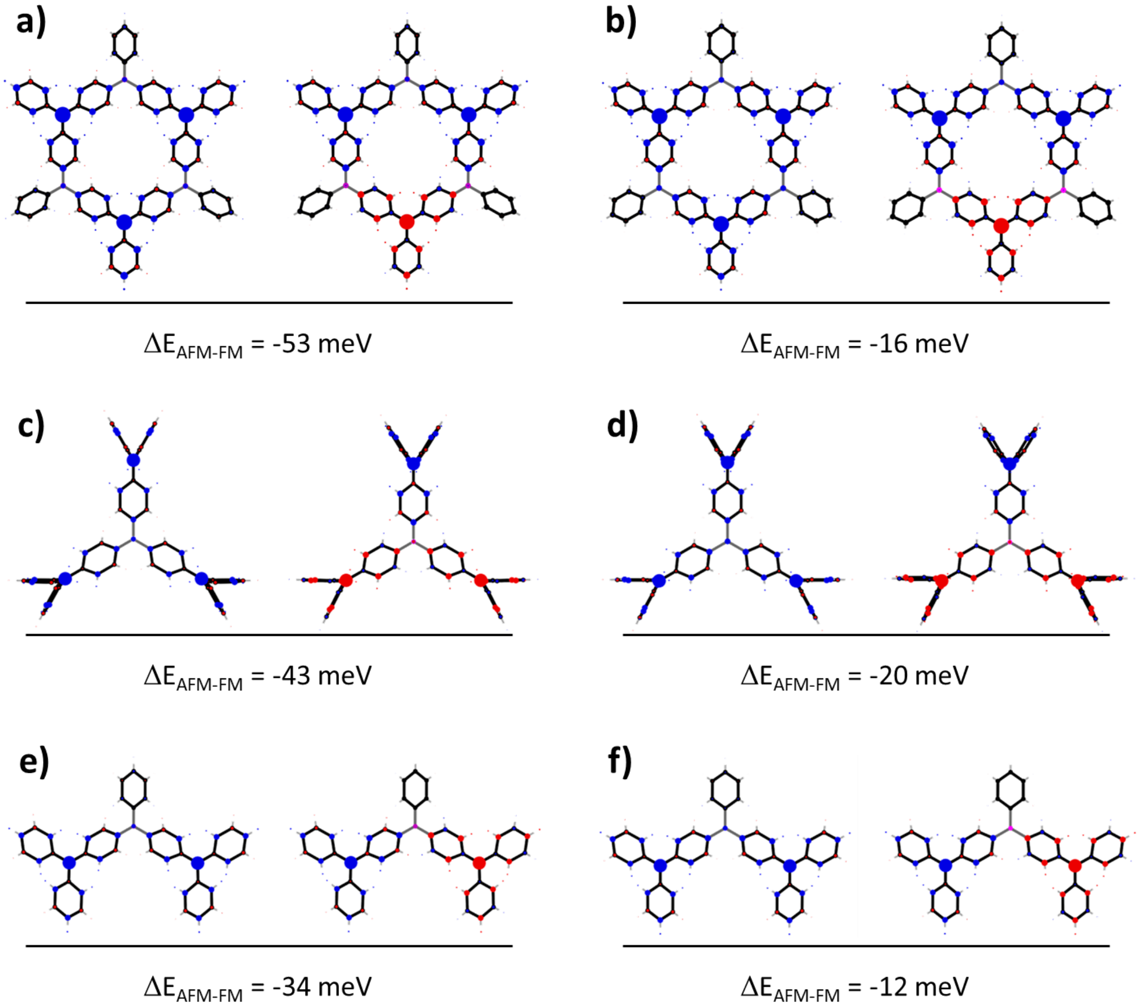

Fig. S4. Atomically-resolved spin population maps for the a) CN- and b) CB-nanorings, the c) CN- and d) CB- models with three magnetic centres and the e) CN- and f) CB- models with two magnetic centres. Both the FM and AFM spin configurations are shown, also providing their energy difference in each case ($\Delta E_{AFM\text{-}FM}$).

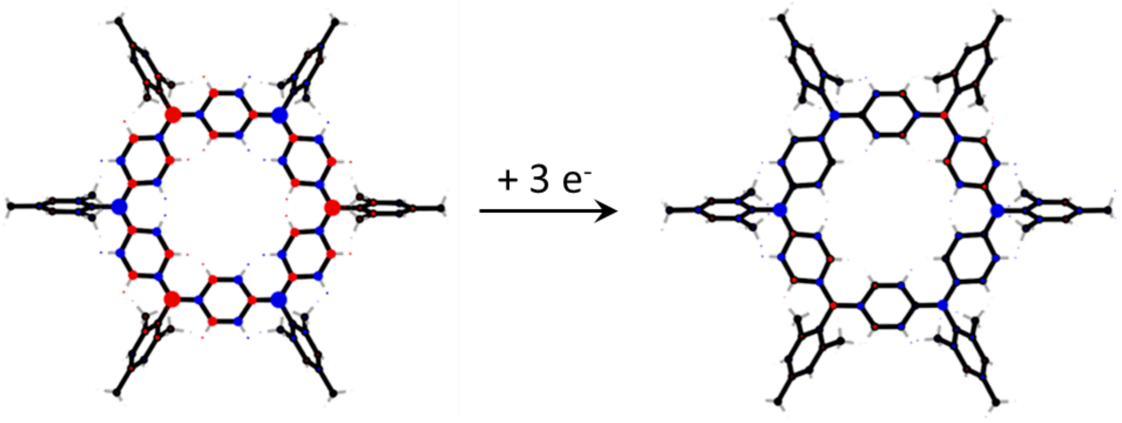

Fig. S5. Atomically-resolved spin population maps for cyclo-para-phenylmethine (analog molecular cycle of C-2DCP) in left: its neutral (AFM) state and right: its 3 e^- reduced state, the latter showing a clear reduction of spin population with no apparent spin localization nor ordering.

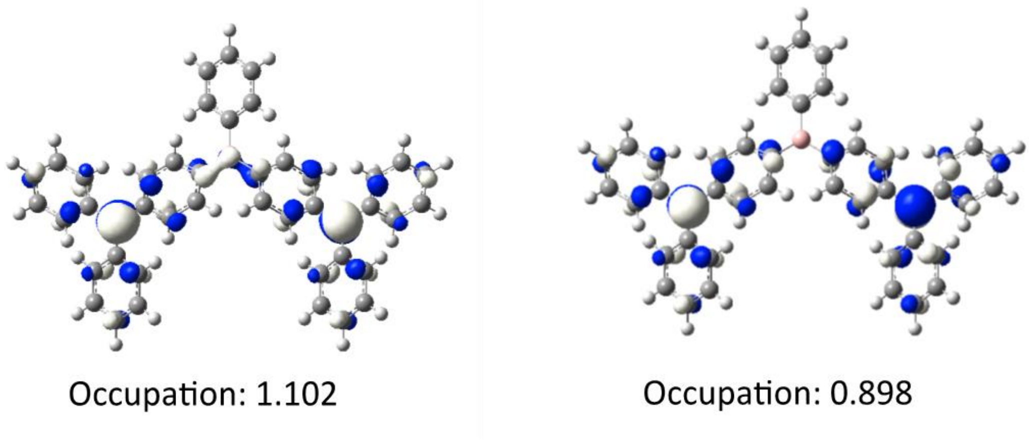

Fig. S6. Singly occupied natural orbitals (SONO) for the singlet ground state of the CB- model with two magnetic centres. These orbitals were obtained from unrestricted DFT calculations using the Gaussian 09 code (Frisch et al. Gaussian 09, Revision B.1; Gaussian, Inc.: Wallingford, CT, 2009.) code using the PBE0 functional and an Ahlrichs TZVP basis set (Schaf̈er et al. J. Chem. Phys. 1994, 100, 5829).

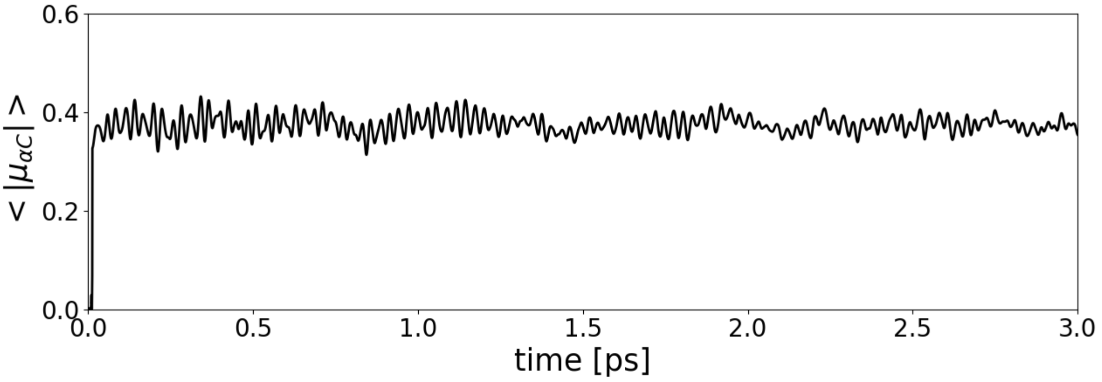

Fig. S7. Average of the absolute atomically-partitioned αC spin population, $\langle|\mu_{\alpha C}|\rangle$, during 3 ps of an *ab initio* molecular dynamics (AIMD) simulation of N-mv-2DCP at 300 K using the PBE0 functional. Details of the AIMD simulations are provided in the Methodology of the main text.

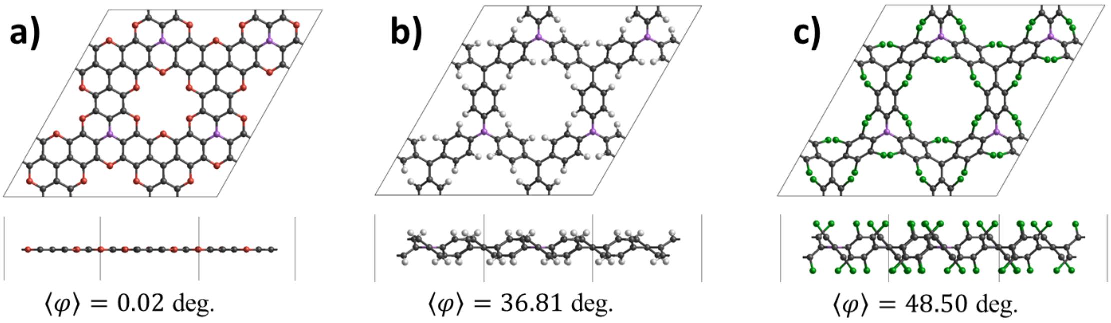

Fig. S8. Z-view (top) and y-view (bottom) of the fully optimized atomic structures (both atomic coordinates and cell parameters) for the ox-N-mv-2DCP, N-mv-2DCP and Cl-N-mv-2DCP. The mean dihedral angle of all aryl rings for each material is provided.

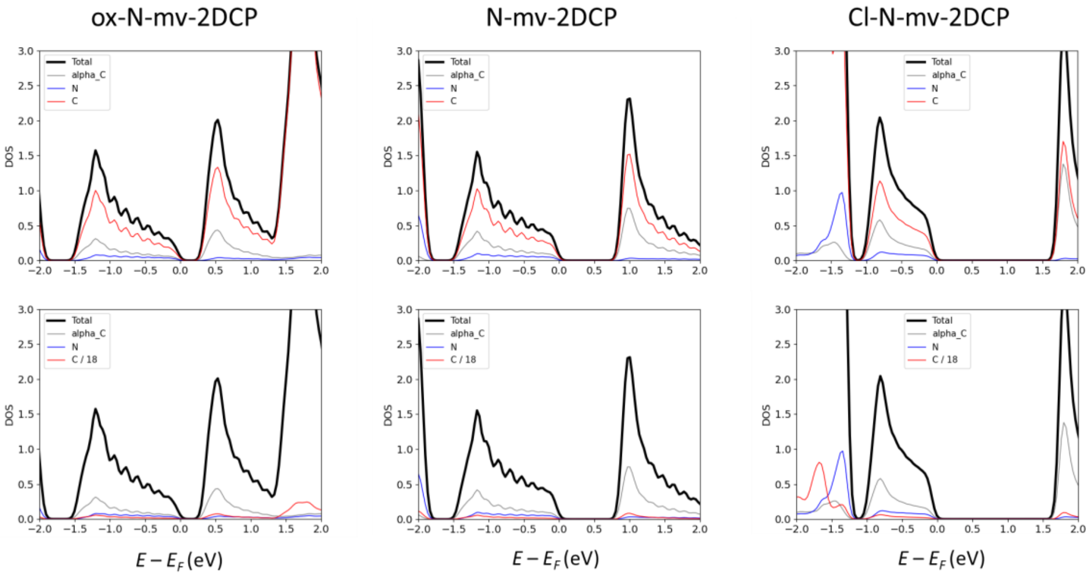

Fig. S9. Species-projected electronic density of states (PDOS) plots for: ox-N-mv-2DCP (left), N-mv-2DCP (middle) and Cl-N-mv-2DCP (right) per unit cell for the FM solution. The upper plots show contributions of all atoms where the contribution from the single N centre (blue), the single αC centre (grey) and the sum of the remaining 18 framework carbon centres (red) have been distinguished. The lower plots show the average contribution per carbon centre from the 18 framework carbon centres (red). In comparison, it is clear that the αC centre makes the single most significant contribution to both the valence and conduction band in each material (grey). Energies are given with respect to E_F which is placed at the top of the valence band.

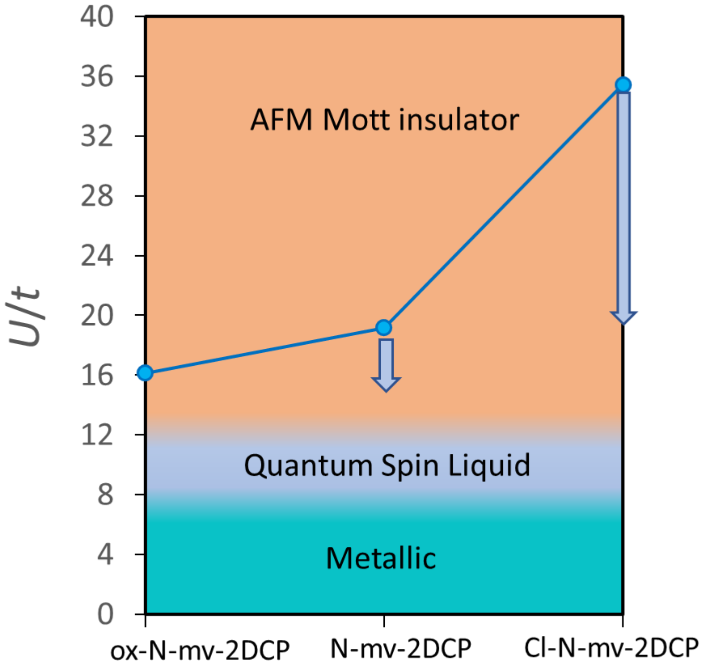

Fig. S10. Estimated variance of U/t in ox-N-mv-2DCP, N-mv-2DCP and Cl-N-mv-2DCP assuming a single band Hubbard model on a 2D triangular lattice (i.e. the emergent lattice of αC centres). The estimated values for the on-site Coulomb repulsion (U) and hopping integral (t) are extracted from our DFT-calculated FM band structures following Xiang et al. *Dalton Trans.* 42, 823 (2013). Specifically, U was derived from the bandwidths of the valence and conduction bands (summed and averaged) and the minimum energy gap in the FM solutions (i.e. $U = W_{av} + E_{gap}$). To estimate t from the FM solutions we used $W_{val}/2z$, where W_{val} is the valence band width and z is the number of nearest neighbours on a triangular lattice (i.e. $z = 6$). The tentative phase assignments are based on those calculated in Szasz et al. *Phys. Rev. X* 10, 021042 (2020) and Chen et al. *Phys. Rev. B* 106, 094420 (2022). Arrows indicate potential decreases in U/t induced by out-of-plane compression on the N-mv2DCP and Cl-N-mv-2DCP materials as estimated from similar effects calculated in Santiago et al. *Adv. Funct. Mater.* 31, 202004584 (2021).

Magnetic coupling constant calculations.

The use of the Heisenberg-Dirac-Van Vleck (HDVV) spin Hamiltonian is justified since most of the spin density is located on the C sp^2 centres thus leading to sites with localised S=1/2 spins. By considering the interactions between pairs of localised S=1/2 spins, the expression we use is:

$$H = \sum_{<i,j>_n} -J_n \cdot \hat{\mathbf{S}}_i \cdot \hat{\mathbf{S}}_j$$

where $\hat{\mathbf{S}}_i$ represents the spin operator on centre *i* and the index $< i, j >_n$ states that the sums runs over all n^{th} neighbour equivalent pairs of spin centres *i* and *j* coupled by the J_n magnetic coupling constants in a given spin system with a defined topology (here, the 2D trigonal or 2D hexagonal topologies are the relevant ones). In the broken symmetry approach, it can be shown (Caballol et al. J. Phys. Chem. A 101 (1997) 7860; Moreira and Illas Phys. Chem. Chem. Phys., 2006, 8, 1645–1659; Rivero, Moreira and Illas J. Phys.: Conf. Series 117 (2008) 012025 doi:10.1088/1742-6596/117/1/012025) that the energy difference between the different electronic solutions can be mapped to the expectation values of the diagonal terms of the HDVV spin Hamiltonian that corresponds to the Ising Hamiltonian given by:

$$H = \sum_{<i,j>_n} -J_n \cdot \hat{S}_i^z \cdot \hat{S}_j^z$$

where $\hat{S}_i^z$ represents the *z* component of the spin operator on centre *i*. Using this mapping approach, the energy differences of the different electronic FM and AFM solutions can be related to the set of coupling constants, J_n, for each of the models described here. To this end, a set of n+1 independent BS solutions are required to extract the different J_n coupling constants considered in the spin model. For the finite spin models used to describe molecular systems the mapping is straightforward. However, for periodic systems some cautions regarding their corresponding energies and expressions to be consistent with the number of chemically equivalent magnetic centres (or formula units, fu) in the unit cell used to calculate the energy differences. In this case, we use the procedure proposed by Rivero, Moreira and Illas (J. Phys.: Conf. Series 117 (2008) 012025 doi:10.1088/1742-6596/117/1/012025) to derive the expressions are extracted by counting the number of interactions assigned to a given unit cell including 1, 2, 3, ... k magnetic centers (or fu). The energy of a given electronic solution corresponding to a particular spin ordering is mapped to the spin model to derive an expression that takes into account one interaction if the pair belongs to the same cell, half of the interaction if each pair is in a different cell (or on a face of the same cell), etc. Electronic solutions obtained with unit cells containing different number of magnetic centres (or fu) can be compared if the corresponding energies and expressions are transformed to the same number of magnetic centres, usually just one formula unit. We have used four different magnetic solutions with two different cells (with three and four fu) to represent the spin orderings in Fig. S11.

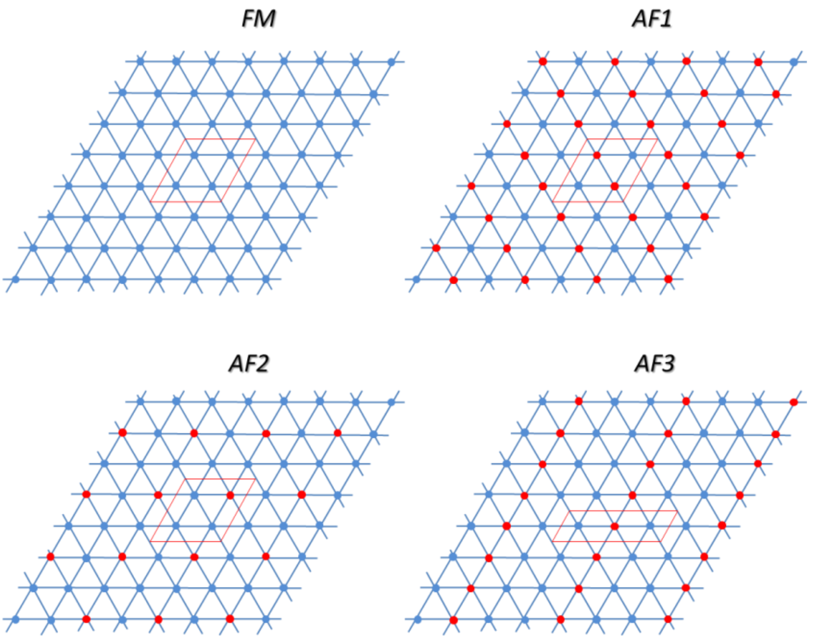

Fig. S11. Magnetic solutions used to extract the magnetic coupling constants described in the text. The energy expressions are provided for each cell and have been used to establish the expressions reported for each energy difference per fu.

The expressions for the energy of these spin orderings are the following ones:

Spin ordering	**Expression per f.u.**
**FM**	$-(3J_1+3J_2+3J_3)/4$
**AF1**	$-(-J_1-J_2+3J_3)/4$
**AF2**	$-(3J_3)/4$
**AF3**	$-(J_1/3-J_2+J_3/3)/4$

From these relations, the set of equations that can be used to extract the values of the magnetic coupling constants are given by

Spin ordering	**Expression relative to FM**	**$E-E_{FM}$/f.u. (meV)**
**FM**	0	0.00
**AF1**	J_1+J_2	-46.22
**AF2**	$(3J_1+3J_3)/4$	-35.62
**AF3**	$(2J_1+3J_2+2J_3)/3$	-32.75

In a first step, we consider the trigonal lattice with the nearest neighbour magnetic interaction only to rationalise the dominant magnetic interaction. In the following table we report the utilized expressions for each model in terms of this approach. In the case of mv-2DCP the magnetic topology leads to a frustrated 2D triangular lattice with equivalent J_1 values to be compared with the cluster model calculations.

	**two magnetic centres model**	**three magnetic centres model**	**nanoring**	**mv-2DCP per f.u.**
**Expression**	$\Delta E_{AFM-FM} = J_1/2$	$\Delta E_{AFM-FM} = J$	$\Delta E_{AFM-FM} = J$	$\Delta E_{AFM-FM} = J_1$

With these expressions we obtain the following values for the nearest-neighbour magnetic coupling constants (J_1):

J_1 / meV	dimer	trimer	nanoring (trimer)	mv-2DCP
**CB-**	-24	-20	-16	-12
**CN-**	-68	-43	-53	-46

The differences between the J_1 values obtained from cluster models with two and three magnetic centres and those obtained from mv-2DCP models arise from significant spin polarisation in the clusters (especially in CB-dimer and CB-trimer clusters). In Fig. S4 the different ΔE_{AFM-FM} values are again provided with the corresponding molecular cluster models.

Even though one can expect that J_1 should be the most important interaction in this system, we also considered the second- and third-next neighbour interactions in the trigonal lattice to analyse the relative magnitude of these coupling constants in the CN-2DCP system. In this case, the values obtained from the system of equations for AF1, AF2 and AF3 gives J_1 =-45.8 meV , J_2 = -0.43 meV and J_3 = -1.57 meV. Similarly, a cluster model with 3 magnetic centers along the path defining J_1 and J_3 results in -60.6 meV and -3.35 meV, respectively. The consistency between these results suggests that the dominant J_1 value is more than one order of magnitude higher than the other magnetic coupling constants and suggests that other non-Heisenberg terms, if present, would play a minor role.

Rationalizing of CN- and CB- differences via molecular orbital diagrams

The qualitative differences in the atomic contributions of the SONOs of the CN- and CB-models can be explained on the basis of a simple molecular orbital (MO) diagram. The MOs that define the magnetic properties of the systems under study result from the combination of the *p* orbitals of the alpha carbons and the *p* orbital of the B or N bridging atom, as shown in Fig. S12. Note that the chemical structure of the diradicals has been coarse grained, that is, only the orbitals of the alpha carbons and the orbital of the bridging atom are shown.

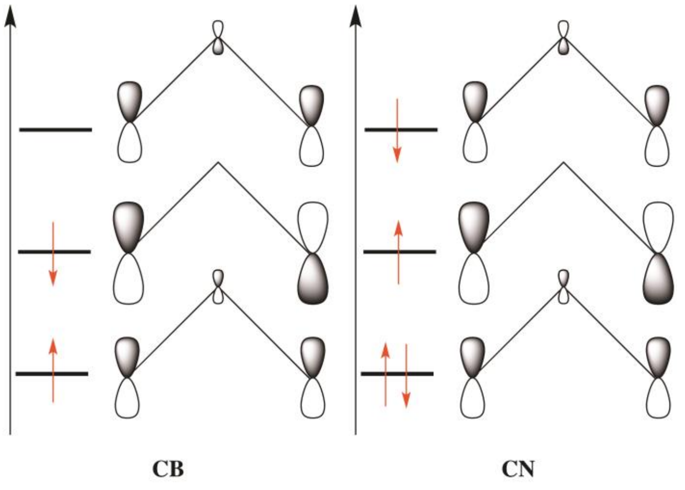

Fig. S12. Simple molecular orbital diagram for the CB (left) and CN (right) models with two magnetic centres.

The most stable MO is the one featuring a bonding combination between the *p* orbitals of the alpha carbons and the *p* orbital of B or N. The less stable MO features an antibonding combination between the *p* orbitals of the alpha carbons and the *p* orbital of B or N. And the MO lying in between is a non-bonding orbital with no contribution of the bridging atom. Given this MO ordering, the number of electrons of the system defines the SOMOs. In the case of the CB system, there are only two electrons because the *p* orbital of B is empty. It then follows that the CB system is a three orbital - 2 electrons system and the SOMOs are the bonding and non-bonding MOs. In the case of the CN system, the *p* orbital of the bridging N atom is filled with 2 electrons, which are added to the electron of each alpha carbon, giving a total of 4 electrons. For this reason, the CN system is a three orbital - 4 electron system and the SOMOs are the non-bonding MO and the anti-bonding MO.
